# Assessment of select electronic health information systems that support immunization data capture – Kenya, 2017

**DOI:** 10.1186/s12913-018-3435-9

**Published:** 2018-08-08

**Authors:** Apophia Namageyo-Funa, Millicent Aketch, Collins Tabu, Adam MacNeil, Peter Bloland

**Affiliations:** 1 Global Immunization Division, Centers for Disease Control and Prevention, 1600 Clifton Road, H24-2, Atlanta, GA 30329 USA; 2 African Field Epidemiology Network, Lugogo House, Ground Floor (Wings B&C), Plot 42, Lugogo By-Pass, P.O. Box 12874, Kampala, Uganda; 3 National Vaccines and Immunization Program, Ministry of Health, P.O Box 43319-00100, Nairobi, Kenya

**Keywords:** Assessment, Data, Electronic health information system, Immunization, Kenya, Registry, Standards

## Abstract

**Background:**

Although electronic health information systems (EHIS) with immunization components exist in Kenya, questions and concerns remain about their use and alignment with the Kenya Ministry of Health’s (MOH) National Vaccine and Immunization Program (NVIP). This article reports on the findings of an assessment of select EHIS with immunization components in Kenya, specifically related to system design, development, and implementation.

**Methods:**

We conducted a rapid assessment of select EHIS with immunization components in Kenya from January to May 2017 to understand the design, development, implementation of the EHIS including the lessons learned from their use. We also assessed how the data elements in the EHIS compared to the data elements in the Maternal and Child Health Booklet used in the existing paper based system in Kenya.

**Results:**

The EHIS reviewed varied in purpose, content, and population covered. Only one system was built to focus specifically on immunization data. Substantial differences in system functionality and immunization-related data elements included in the EHIS were identified. None of the EHIS had all the data elements necessary to fully replace or operate independently from the standardized paper-based system for recording immunization data in Kenya.

**Conclusions:**

Overall, the findings of this assessment highlighted substantial variation in the EHIS with immunization components. The findings provide insights and lessons learned for the Kenya MOH NVIP, immunization partners, vendors of EHIS, and users of EHIS to consider as Kenya transitions from paper-based to electronic immunization information systems.

## Background

In Kenya, routine immunization services are primarily documented and the information stored at the point of service, usually health facilities, using standardized paper-based immunization information systems (IIS). With the recent advances in technology, global interest is growing to transition from standardized paper-based to electronic IIS at all levels of the health service system. The benefits of an electronic IIS include an efficient way to 1) collect immunization data systematically, 2) access and retrieve immunization data easily, and 3) analyze, report, and facilitate use of immunization data for public health decision-making [1–3]. The Kenya Ministry of Health (MOH) has an interest in expanding use of electronic health information systems (EHIS) [4], as evidenced by the EHIS that have been introduced in Kenya for various health related functions such as management of data on maternal and child health [5]. The Kenya MOH National Vaccine and Immunization Program (NVIP) is also interested in moving from the use of paper-based systems to electronic IIS.

A move from paper to electronic IIS would support use of immunization data to guide the NVIP goal of reducing morbidity, mortality, and disability from vaccine preventable diseases. For an electronic IIS to be useful to NVIP, a number of discussions about the system should be held with relevant stakeholders on the purpose and the objectives of the system, how it should be designed, what type of data it should collect and so forth. The discussions ensure the design, development, and use of a system align with the system’s intended purpose and NVIP goal. To facilitate the use of immunization data by NVIP, the electronic IIS should include immunization specific standards. Immunization specific standards are useful for supporting access to individual immunization status, forecasting recommended vaccines, immunization coverage reporting, adverse effects following immunization(AEFI) reporting, interoperability with other systems, and improved defaulter tracking and reduction of delayed vaccinations. NVIP can also use the immunization specific standards to facilitate the larger country effort discussions to strengthen and harmonize the integration of immunization systems with existing health systems in Kenya.

Although EHIS with immunization components exist in Kenya, questions and concerns remain about their potential use and alignment with the Kenya NVIP mission: for example, do the EHIS produce immunization-specific NVIP required reports; do the EHIS support tracking of defaulters and those lost to follow up; and can the EHIS share information with each other? The EHIS that capture and report immunization data function either as a stand-alone system or via an immunization-specific data module added to an existing system. Furthermore, no public documentation is available on how the EHIS compare in terms of functionality, data elements, workflow, or capacity to share information with other EHIS. This article reports on the findings of a rapid assessment of select EHIS with immunization components in Kenya. The assessment provides lessons learned to guide future efforts on the design, development and implementation of EHIS to support the immunization needs of the NVIP and Kenya.

## Methods

We conducted a rapid assessment of select EHIS with immunization components to understand the design, development, implementation of the EHIS including the lessons learned from their use. We also assessed how the data elements in the EHIS compared to the data elements in the Maternal and Child Health Booklet used in the existing paper based system in Kenya.

### Setting for assessment activities

The assessment occurred in Kenya from January to May 2017 at the offices and health facilities associated with the use of the respective EHIS selected for the assessment. Kenya is a country located in East Africa and that has a vested interest in the use of EHIS as evidenced by the more than 16 EHIS in the country [5] and the Kenya national electronic health (e-health) strategy 2011–2017 that has a focus on health information systems [4]. Examples of the EHIS include International Quality Care and Kenya Electronic Medical Record, to name a few [5]. Kenya has also developed resources and tools such as the Standards and Guidelines for Electronic Medical Record (EMR) Systems in Kenya [6] and the Standards and Guidelines for primary health care EMR systems in Kenya [7], for use by vendors, developers, and implementers of EHIS in Kenya. The continued interest in promoting electronic systems in Kenya is further reflected in the new document on the national electronic health strategy for the years 2016 to 2030 [8].

### Selection of electronic health information systems

Given the purpose of the rapid assessment was to examine a few health systems, the MOH NVIP identified a convenience sample of EHIS to include in the assessment. Criteria for inclusion in the assessment included an EHIS that collects immunization data, an EHIS that is in use by immunization staff, and EHIS management interest in participating in the assessment. We contacted staff engaged in the design, implementation, or evaluation of the identified EHIS by phone and email to confirm the existence and use of the system for collecting immunization data and to invite them to participate in the assessment.

### Selection of staff and health facilities

Selection of and the number of staff and health facilities to engage in the assessment was at the discretion of the EHIS management team. County Health Managers, system managers, health facility in-charges, health care workers, nurses, data entry clerks, Health Record and Information Officers, and Information and Communication Technology officers were examples of staff invited to participate in the assessment. Criteria for participation included staff who are engaged in the design, implementation, or evaluation of the EHIS. We also included at least two health facilities per EHIS to participate in the assessment. Criteria for inclusion in the assessment included a health facility that had the EHIS on site and that had staff using the EHIS.

#### Data collection

We used a semi-structured questionnaire to collect information on the systems design, development, implementation of the EHIS including the lessons learned from their use. The semi-structured questionnaire included questions related to describing the EHIS, guidance used to develop the EHIS, the EHIS functionality, data elements, work flow, data entry process, the process of unique identifier generation, protection of privacy, data confidentiality, data sharing/exchange, capacity building efforts, and sustainability efforts implemented. The assessment also comprised field visits to one or more health facilities with the EHIS to observe use of the system by staff and to gather information on the barriers and facilitators to use of the EHIS. Observations from the field also served to validate the information obtained from speaking with staff not located at the health facility. The duration of the field visits was between 1 to 2 h depending on health facility. This length of time was to ensure minimal interruption to the ongoing activities at the health facility while we conducted the field visits.

#### Data analysis

For each EHIS we compiled the data describing the EHIS, guidance used to develop the EHIS, the EHIS functionality, data elements, work flow, data entry process, the process of unique identifier generation, protection of privacy, data confidentiality, data sharing/exchange, capacity building efforts, and sustainability efforts implemented. When possible, we cross verified the responses from staff with the data from the observations. For all the EHIS we analyzed the notes on the barriers and facilitators to generate themes related to the use of the EHIS. To assess how the EHIS compared to the existing standardized immunization paper-based system in Kenya, the immunization-related data elements used within each EHIS were compared to the data elements included in one of the more than six tools used in the standardized paper-based immunization forms used across Kenya – the Maternal and Child Health Booklet (MCHB). This assessment did not examine the quality of the data within the EHIS.

#### Ethical review

This assessment was granted exemption from requiring ethics approval by the Centers for Disease Control and Prevention Institutional Review Board protocol # 2018–037. The assessment activities were limited to public health program evaluation to inform future decisions about the use of EHIS in Kenya. Written consent for EHIS staff to participate was not required because data was collected on the characteristics and use of the EHIS and not on individually identifiable information on the EHIS staff.

## Results

### System description

All six EHIS assessed were established within the past decade; the oldest was developed in 2008 (10 years old) and the most recent in 2015 (Table 1). Only one of the EHIS focused solely on immunizations specifically while five collected broader data, primarily on HIV/AIDS or maternal and child health. Generally, the target population covered by the EHIS was the catchment population of the specific health facility; however, specific target population groups were the focus of some EHIS. For example, one EHIS (EHIS 2) focused only on children under 18 years of age whereas two focused on populations living with HIV/AIDS (EHIS 4 and 5); the latter provided immunization services only to children with HIV infection or children with HIV-infected mothers.

**Table 1:** Characteristics of select health facility-based Electronic Health Information Systems in Kenya

EHIS characteristic	EHIS 1	EHIS 2	EHIS 3	EHIS 4	EHIS 5	EHIS 6
Age of system in years	10	7	3	10	7	2
Number of counties covered	1	4	1	22	22	5
Number of health facilities participating	34	13	7	470	353	75
Primary purpose is immunization record keeping	✓					
Includes outreach immunization activities	✓					
Is part of a hospital-wide system		✓	✓	✓		✓
Web-based access	✓	✓	✓			
Local Area Network access				✓	✓	✓
Technical support on site	✓	✓	✓			
System software is open source			✓	✓	✓	✓

Five of the EHIS operated within MOH-run health facilities while one EHIS (EHIS 2) operated only in private health facilities. Four of the five EHIS operating within MOH-run facilities required existing health care workers to enter data into the EHIS in addition to their routine tasks, while one system (EHIS1) used someone specifically hired to enter data.

All six EHIS used both electronic and paper data entry. The paper data entry was limited to registration for immunization, anthropometric measurement, and recording of vaccination events in the MCHB. All EHIS were designed using program-specific guidance (e.g., National AIDS and STI Control Program requirements) and MOH approved data collection and reporting tools (e.g., the Kenya Expanded Program on Immunization [KEPI] vaccination schedule as determined by the NVIP), then further streamlined based on feedback from users of EHIS and observations in the field.

### System functionality and data elements

Although all six EHIS captured immunization data, there was variation in the functionality and the corresponding way immunization-related data elements were collected. All EHIS were designed to: 1) use passwords and role-based access to facilitate privacy and confidentiality of information, 2) automatically generate individual-level unique identifiers, 3) capture basic demographic information on mothers and children, 4) capture basic immunization data (vaccines required and received according to the KEPI vaccination schedule), and 5) generate monthly programmatic reports. None of the EHIS had functionality to link to District Health Information System version 2 or to generate MOH reports specific to immunization such as the Immunization Services Uptake Summary Report – MOH 710 Summary Report.

Other functions that were observed in the six EHIS included ability to capture information related to AEFI (*n* = 1), monitor and manage vaccine stocks (*n* = 2), send text message reminders to parents (*n* = 1), capture additional vaccines administered beyond those in the KEPI vaccination schedule (*n* = 1), identify fully immunized children at the individual level (*n* = 1), and identify missed opportunities and defaulters at the individual level (*n* = 1).

Demographic information including the unique identifier, vaccines administered according to the KEPI vaccination schedule, and date/time vaccine administered were data elements from the MCHB captured in all EHIS (Table 2). There was variation in the capture of other MCHB data elements such as site of vaccine administration and list of additional vaccines within the six EHIS. None of the EHIS had all of the immunization related data elements of the existing standardized paper-based system for immunizations in Kenya.

**Table 2:** Comparison of selected data elements in Electronic Health Information Systems and Maternal Child Health Booklet

Data element	EHIS 1	EHIS 2	EHIS 3	EHIS 4	EHIS 5	EHIS 6
Child level data						
Name of child	✓	✓	✓	✓	✓	✓
Date of birth	✓	✓	✓	✓	✓	✓
Sex	✓	✓	✓	✓	✓	✓
Birth order	✓					
Unique identifier of child	✓	✓	✓	✓	✓	✓
Anthropometrics	✓	✓	✓	✓	✓	✓
Health facility information						
Permanent register number				✓		
Health facility number/name	✓	✓	✓	✓		✓
Master facility list number			✓			✓
Name of the vaccine administrator	✓	✓	✓	✓		✓
Immunization services						
List of vaccines in KEPI^a^ vaccination schedule	✓	✓	✓	✓	✓	✓
List of vaccines not included in KEPI^a^ vaccination schedule		✓				
Date and time vaccine is administered	✓	✓	✓	✓	✓	✓
Site of vaccine administration		✓	✓		✓	
Vaccine amount/dose		✓	✓		✓	✓

^a^Kenya Expanded Programme on Immunization

### Facilitators and barriers

The facilitators and barriers on the use of the EHIS are reported in Table 3. All the facilitators reported were related to EHIS in general. None of the facilitators were specific to the immunization components of the EHIS. Factors that facilitated use of EHIS were related to users (e.g., presence of an EHIS champion – an individual interested in promoting the use of EHIS, available technical support for the system) and to system functionality (e.g., ability to access and retrieve patient data easily).

**Table 3:** Facilitators and barriers to use of select Electronic Health Information Systems

Facilitators of EHIS use:	
- Ability to easily access and retrieve individual patient data - Ability to easily manage aggregate data within the EHS - Health care worker involvement in development of EHIS - Ability to receive feedback from EHIS users - Availability of technical support on the EHIS for users of the EHIS - Frequent refresher trainings for users of the EHIS - An EHIS champion to promote use of the EHIS - Ability to backup data (backup server and internet provider)	
Barriers to EHIS use:	
- Power outages - Slow internet connectivity - Staff shortages - Knowledge gaps among users - Time needed for data entry - Double entry of data on paper and in the EHIS - Inability to update/include previously given vaccines - Inability to generate reports required by the National Vaccines and Immunization Program	

Some barriers were related to issues not specific to an individual EHIS (e.g. power outages) while other barriers related to immunization-specific aspects of EHIS (e.g., inability to generate the required NVIP immunization reports). Barriers were also related to users (e.g., slow adoption of EHIS by facility staff, gaps in knowledge about use of the electronic system) and to system functionality (e.g., inability to update/include previously given vaccines).

## Discussion

From the six EHIS reviewed, only one system was built to focus specifically on immunization data. The one EHIS was also the only one focused on the inclusion of outreach data on immunization. This finding supports the notion of leveraging existing EHIS to incorporate immunization components. In this assessment, the reach (immunization and non-immunization) of these existing systems (EHIS 4 with 470 participating facilities and EHIS 5 with 353 participating facilities) was high which shows the potential to reach many more children than the other EHIS examined in this assessment. This would be especially be the case if the EHIS expanded their target population to include populations other than children with HIV only as was reported in this assessment. The NVIP could also advocate for the expansion of the purpose of the system and inclusion of components such as outreach that are important for the planning and delivery of immunization services. Expansion of the purpose of the system could facilitate the use of the system for immunization in addition to the other purposes designed for the system. Future assessments should explore the benefits to immunization programs of standalone versus integrated electronic IIS.

Although this assessment only looked at EHIS in comparison to one of the standardized paper-based system tools – the MCHB, our findings showed that none of the EHIS had all the data elements necessary to fully replace or operate independently from the standardized paper-based system for tracking immunizations in Kenya. Perhaps the use of a hybrid model of both paper and electronic IIS may be the next step in the future of data collection and reporting in Kenya. As countries transition from standardized paper-based systems to electronic IIS, there is a desire for the data collected in the former to also be collected in the latter. Given the challenges of staffing capacity and workload in the implementation of EHIS [9, 10] consideration should be given to what data needs to be collected in both paper and electronic IIS. In this assessment for all but one system, health facility staff were in charge of data entry, adding to staff work burden that could impact data completeness and quality from the EHIS. Although our assessment did not look at data quality, the findings suggest a need for periodic assessments of the quality of immunization data captured by EHIS.

The assessment showed substantial differences in the functionality and immunization-related data elements included in the EHIS, which might reflect a lack of nationally endorsed immunization specific standards for electronic IIS. The lack of immunization-specific standards and data elements limited the usefulness of these systems, especially concerning the ability to share data with national-level health information systems. In some countries (e.g., United States, Canada) immunization specific standards for electronic IIS are available to promote consistent data collection and reporting processes [11, 12]. The immunization specific standards also guide the development of electronic IIS to ensure alignment with government information policies and relevant data requirements on immunization. Although Kenya has guidance on standards for EMRs [6, 7], the guidance primarily focuses on HIV-related information with a few sections for immunization content in electronic systems in the “*Standards and guidelines for primary health care electronic medical record systems in Kenya”* [7]. Development of immunization specific standards and minimum data elements consistent with the goals of NVIP, and aligned with other national health information systems, would greatly facilitate collection and reporting of immunization data in Kenya.

The facilitators and barriers reported in this assessment are similar to those reported in the literature on implementation of EHIS generally. A systematic review among users in developed countries reported time, cost, and technical support as factors related to the successful use of EHIS [13]. Reviews and studies focused on developing countries found that internet connectivity, availability of electricity, computer literacy, staffing capacity, data quality, and availability of standard operating procedures for the systems affected the implementation of EHIS [9, 10]. NVIP should consider the facilitators reported to enhance the use of EHIS once the country is ready to transition from standardized paper-based systems to electronic IIS. Only two barriers reported related to immunization specific issues of an EHIS – inability of the EHIS to update/include previously given vaccines and inability of the EHIS to generate reports required by NVIP. Development of immunization specific standards on data to be collected in an EHIS and type of reports to include can help address the two barriers. Addressing the other barriers to the use of EHIS reported in this assessment can include reference to efforts on EHIS in general and not specific to immunization related EHIS e.g. power outages and staff shortages.

This rapid assessment had limitations. The interviews included in this assessment were all self-reports, and responses from staff for the different EHIS might have been subject to bias. Similarly, given the open-ended format of certain questions, interviewer bias could have been introduced as interviews proceeded. This assessment only included EHIS identified by the Ministry of Health NVIP. Consequently, the sample might not have included all EHIS with immunization components in Kenya. We reached out to all the EHIS that the NVIP was aware of and all agreed to participate in this assessment. The health facilities we visited were identified by the management of the EHIS, which might have influenced the findings on the barriers and facilitators to use of the EHIS.

## Conclusions

Overall, the findings of this assessment highlight substantial variation in the EHIS with immunization components and provide lessons learned for the Kenya MOH NVIP, immunization partners, vendors of EHIS, and users of EHIS to consider as Kenya considers the transition from paper-based to electronic IIS. Is a hybrid version of both paper and electronic IIS best for Kenya? Which is more beneficial for Kenya, standalone or integrated IIS? Development of immunization specific standards for electronic IIS that align with those of other national EHIS is one way that might help reduce variation in the systems as newly developed electronic IIS are introduced in Kenya. The immunization specific standards will allow for sharing of data across systems if one system is not an option for Kenya.

## Abbreviations

AEFI: Adverse Event Following Immunization
EHIS: Electronic Health Information System
EMR: Electronic Medical Record
IIS: Immunization Information System
KEPI: Kenya Expanded Program on Immunization
MCHB: Maternal and Child Health Booklet
MOH: Ministry of Health
NVIP: National Vaccine and Immunization Program
